# Intrapersonal and interpersonal processes of social exclusion

**DOI:** 10.3389/fnins.2015.00062

**Published:** 2015-03-06

**Authors:** Taishi Kawamoto, Mitsuhiro Ura, Hiroshi Nittono

**Affiliations:** ^1^Japan Society for the Promotion of ScienceTokyo, Japan; ^2^Faculty of Integrated Arts and Sciences, Hiroshima UniversityHigashi-Hiroshima, Japan; ^3^Department of Psychology, Otemon-Gakuin UniversityIbaraki, Japan

**Keywords:** social exclusion, intrapersonal process, interpersonal process, fMRI, ERPs

## Abstract

People have a fundamental need to belong with others. Social exclusion impairs this need and has various effects on cognition, affect, and the behavior of excluded individuals. We have previously reported that activity in the dorsal anterior cingulate cortex (dACC) and right ventrolateral prefrontal cortex (rVLPFC) could be a neurocognitive index of social exclusion (Kawamoto et al., 2012). In this article, we provide an integrative framework for understanding occurrences during and after social exclusion, by reviewing neuroimaging, electrophysiological, and behavioral studies of dACC and rVLPFC, within the framework of intrapersonal and interpersonal processes of social exclusion. As a result, we have indicated directions for future studies to further clarify the phenomenon of social exclusion from the following perspectives: (1) constructional elements of social exclusion, (2) detection sensitivity and interpretation bias in social exclusion, (3) development of new methods to assess the reactivity to social exclusion, and (4) sources of social exclusion.

## Introduction

Humans are abundantly social animals, and have a fundamental *need to belong* (Baumeister and Leary, 1995). Maintaining good and lasting relationships with others is therefore quite vital for us (Baumeister and Leary, 1995; Macdonald and Leary, 2005). **Social exclusion** breaks the relationships we have with others and influences our physical and mental health in a wide range of ways. For example, previous studies have revealed that social exclusion possibly promotes suicide (Van Orden and Joiner, 2013), increases depression (Nolan et al., 2003), and even decreases survival rates in mammals generally (Kling et al., 1970; Silk et al., 2003) and humans specifically (Holt-Lunstad et al., 2010). Social exclusion also causes aggression in humans (e.g., Twenge et al., 2001; Warburtona et al., 2006; Gaertner et al., 2008; DeWall et al., 2009b; Wesselmann et al., 2010) and is thought to be one of the causes of school shootings (Leary et al., 2003). Furthermore, social exclusion is not rare or unusual in daily life events (Nezlek et al., 2012), and has the potential to occur in a variety of places including text messages on cell phones (Smith and Williams, 2004) and on Facebook (Tobin et al., 2015).

KEY CONCEPT 1 **Social exclusion**

In this article, we refer to social exclusion as events and situations that signal a lack of social connections with others. Therefore, this article includes studies referring to ostracism, devaluation, and social rejection.

**Figure 1:**
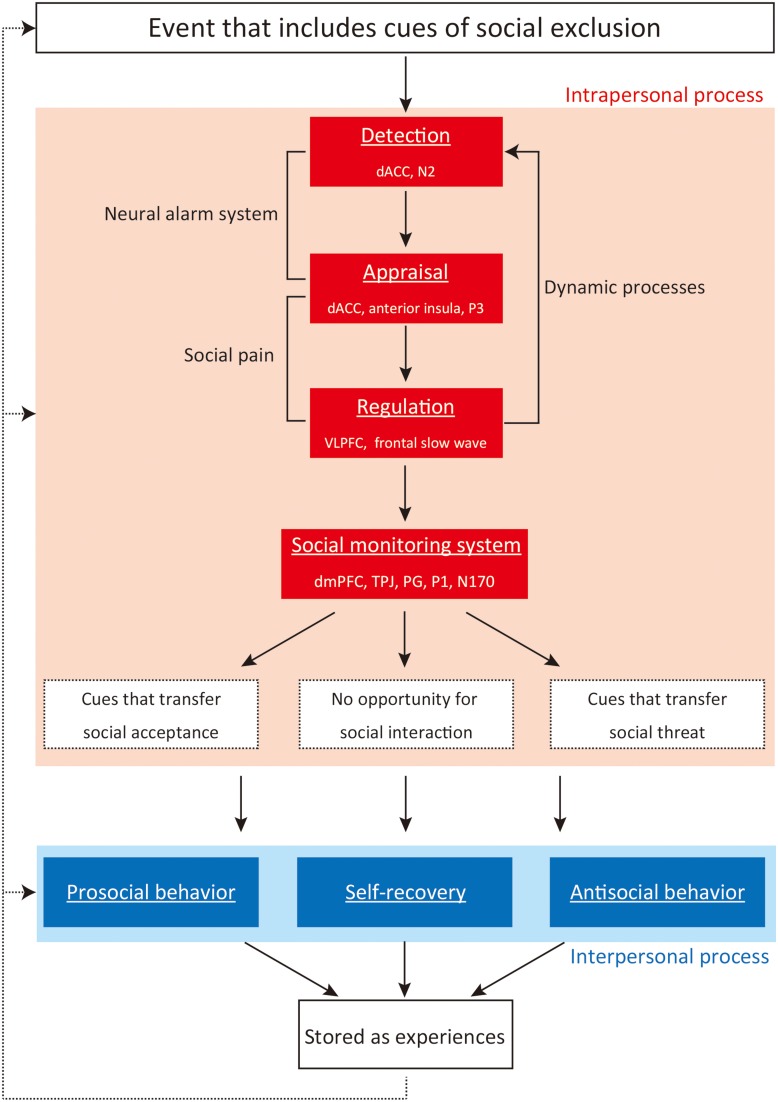
**Intrapersonal and interpersonal processes of social exclusion**. Note: dACC, dorsal anterior cingulate cortex; VLPFC, ventrolateral prefrontal cortex; dmPFC, dorsomedial prefrontal cortex; TPJ, temporoparietal junction; PG, precentral gyrus.

In light of the severe and wide ranging effects of social exclusion on the psychological adaptation of individuals, it is important to identify neural correlates, cognitions, and behaviors that occur during and after social exclusion. We have previously investigated the intrapersonal process of social exclusion by using functional magnetic resonance imaging (fMRI), and identified neural correlates of social exclusion by comparing social exclusion and expectancy violation. As reported in *Frontiers in Evolutionary Neuroscience* (Kawamoto et al., 2012), we identified dorsal anterior cingulate cortex (dACC) and right ventrolateral prefrontal cortex (rVLPFC) activities as a possible neurocognitive index of social exclusion. In this focused review article, we have reviewed studies on the involvement of these two brain regions in intrapersonal and interpersonal processes of social exclusion. We provide an integrative framework for understanding *intrapersonal and interpersonal processes of social exclusion* (Figure 1), and review the literature of social exclusion using neuroimaging, electrophysiological, and behavioral methods within this framework. First, we review three stages—detection, appraisal, and regulation—which occur during social exclusion. Second, we review how social exclusion changes perception, attention, and cognition in response to social cues (social monitoring system: SMS). Third, we review how people behave following social exclusion and the way they recover from the aversive impact of social exclusion (interpersonal processes). Fourth, we review how intrapersonal and interpersonal processes of social exclusion are stored as experiences, and how these influence responses to future social exclusion. Finally, we suggest some directions for future studies that we believe are needed for a better understanding of social exclusion.

## Intrapersonal processes during social exclusion: detection, appraisal, and regulation

Table 1 summarizes the key studies that have investigated the role of intrapersonal processes of social exclusion. People can detect social exclusion in any situation (Williams, 2009; Wesselmann et al., 2012a). Previous research using “Cyberball” software has revealed that we feel excluded even if our excluders are online (Williams et al., 2000), computer-generated opponents (Zadro et al., 2004; Kawamoto et al., 2013b), or disliked others (Gonsalkorale and Williams, 2007). In the Cyberball paradigm, participants play a simple ball-tossing game with two or three opponents through the computer screen. In the exclusion condition, participants experience social exclusion by receiving few ball tosses from others. Individuals feel **social pain** during social exclusion (Eisenberger et al., 2003). Prior research has investigated the neural correlates of detection, appraisal, and regulation processes of social exclusion. Three brain regions—dACC, anterior insula (AI), and VLPFC—play a particularly important role in cognitive and affective processing during social exclusion. In a highly influential study, Eisenberger et al. (2003) conducted an fMRI experiment to identify the brain regions associated with processing social exclusion during Cyberball play. They found that the dACC, AI, and right VLPFC were more activated during the social exclusion condition relative to the fair play condition (i.e., when participants caught the ball equally as often as the other players). In addition, dACC activation was positively correlated with the amount of social pain participants felt during social exclusion, whereas right VLPFC activation showed the opposite pattern.

**Table 1:** **Key studies on intrapersonal processes of social exclusion**.

**References**	**Tools**	**Key findings**
Bolling et al., 2011b	fMRI	dACC and ventral ACC (vACC) showed increased activation in response to social exclusion as compared to rule violations.
Crowley et al., 2009	ERP	Frontal slow waves were related to the regulation of social pain.
Cacioppo et al., 2013	Meta-analysis	The results failed to show a significant involvement of dACC in response to social exclusion, suggesting that social exclusion could be more complex than previously thought.
Eisenberger et al., 2003	fMRI	dACC was positively related to social pain whereas rVLPFC was negatively related to social pain.
Kawamoto et al., 2012	fMRI	dACC and rVLPFC showed increased activation in response to social exclusion after controlling for expectancy violations.
Rotge et al., 2015	Meta-analysis	dACC and vACC were involved in processing of social exclusion.
Somerville et al., 2006	fMRI	dACC was sensitive to expectancy violations whereas vACC was sensitive to social scenarios.
Themanson et al., 2013	ERP	N2 was sensitive to conflict monitoring during social interactions whereas P3b was related to social distress in response to social exclusion. Both ERP components decreased with time.
Williams et al., 2000		Online exclusion caused a decrease of fundamental needs (i.e., self-esteem, belonging, meaningful existence, and control).

KEY CONCEPT 2 **Social pain**

The distressing experiences arising from the perception of actual or potential psychological distance from close others, or social groups (Eisenberger and Lieberman, 2004, p. 294). We often use the term “painful” when we are referring to social exclusion and social exclusion is known to activate brain regions similar to those that are activated during physical pain (e.g., dACC).

We often use the term “painful” even when we are referring to non-physical injuries. In fact, the use of the words pain and painful to describe when individuals are being excluded from partner or group relationships is common across many different languages (Macdonald and Leary, 2005). Social pain has been defined as “the distressing experience arising from the perception of actual or potential psychological distance from close others or a social group (Eisenberger and Lieberman, 2004, p. 294).” Social pain is more than just a metaphor as it shares common neural correlates with physical pain (Eisenberger and Lieberman, 2004; Macdonald and Leary, 2005; Eisenberger, 2012a,b, 2015). In fact, social exclusion activates the similar brain regions that have been associated with appraisal—the dACC and AI—and regulation—the VLPFC—of the unpleasantness of physical pain (e.g., Eisenberger et al., 2003; Eisenberger and Lieberman, 2004; Eisenberger, 2012a,b, 2015). In addition, previous studies have revealed the parallel nature of increasing physical and social pain (DeWall and Baumeister, 2006; Eisenberger et al., 2006), further supporting their commonality. Thus, both the dACC and Al seem to be related to appraisal of social exclusion whereas the VLPFC seems to be related to regulation of social pain evoked by social exclusion.

Although dACC involvement in processing social exclusion has been replicated by multiple studies (e.g., Eisenberger et al., 2007, 2011; Onoda et al., 2009, 2010; DeWall et al., 2012), social exclusion is a complex phenomena which inherently includes multiple other components such as **expectancy violation** Previous studies have been challenged to differentiate social exclusion and mere expectancy violation (Somerville et al., 2006; Bolling et al., 2011b; Kawamoto et al., 2012). Our group compared brain activation during social exclusion and during over-inclusion—participants received many throws—using Cyberball (Kawamoto et al., 2012). We found that the dACC was more activated for exclusion-related events than over-inclusion-related events, but failed to show any relation between the dACC and self-rated social pain. On the other hand, rVLPFC was negatively correlated with social pain, supporting the notion of the regulatory function of rVLPFC on social pain (Eisenberger et al., 2003). Note that we did not observe any significant relation between the dACC and self-reported expectancy violation scores (e.g., surprise feeling), suggesting that dACC activation in response to social exclusion is not merely due to expectancy violation. Other studies have found a role of the dACC in expectancy violation (Somerville et al., 2006), and both social exclusion and expectancy violation (Bolling et al., 2011b). How do we interpret this seemingly inconsistent evidence? One possible explanation is to conceptualize dACC function in social exclusion as a “**neural alarm system**” (Eisenberger and Lieberman, 2004). Eisenberger and Lieberman argued that detection and appraisal processes involved in the dACC were complementary processes underlying the function of the neural alarm system. Therefore, the dACC plays an important role in both detection and appraisal of social exclusion.

KEY CONCEPT 3 **Expectancy violation**

A situation when individuals' expectations are violated. In exclusion studies using Cyberball, participants are often included before being excluded, resulting in expectations of social inclusion during social exclusion. Thus, some research has argued that responses to social exclusion not only include painful experiences but also cognitive conflict that comes from expectancy violation.

KEY CONCEPT 4 **Neural alarm system**

Neural systems that signal relational threats. This system was proposed in order to explain the role of the dACC in response to social exclusion. Two sub-systems are considered to be needed for adequate operation of the neural alarm system. The first is a discrepancy monitoring system, which serves to detect deviations from desired standards. The second is a sounding mechanism that signals a problem that needs to be addressed. The discrepancy detection function is considered to be associated with the detection of social exclusion, whereas social pain is thought to be the product of the sounding system.

However, results of meta-analyses have been inconclusive. One recent meta-analysis of fMRI studies related to social exclusion showed the involvement of dACC in processing information related to social exclusion (Rotge et al., 2015), whereas another meta-analysis failed to show an association between social exclusion and dACC (Cacioppo et al., 2013). These contrary findings suggest that neural correlates of social exclusion might be complex (Cacioppo et al., 2013), and need further investigation focusing on the duration and temporal dynamics of social exclusion (Rotge et al., 2015). Supporting this notion, recent electrophysiological and neuroimaging studies have suggested that intrapersonal processes of social exclusion dynamically change with time, and occur not only in whole exclusionary situations but also during specific events within social interactions (e.g., Crowley et al., 2009, 2010; Kawamoto et al., 2012, 2013b; Moor et al., 2012; Themanson et al., 2013). For example, prior findings using event-related brain potentials (ERPs) indicated that N2, P3b, and frontal slow wave in response to each exclusionary cue (i.e., throws among opponents in Cyberball) were closely related to the detection, appraisal, and regulation processes of social exclusion, respectively (Crowley et al., 2009, 2010; Kawamoto et al., 2013b; Themanson et al., 2013). In addition, our group found that attention decreased with time, negative affect accumulated with time, and motivation shifted to a withdrawal pattern during social exclusion (Kawamoto et al., 2013b). Given the previous findings, it is possible that individuals repeatedly process each exclusionary cue, and additive and complex intrapersonal processes determine the final emotional response (e.g., social pain). Thus, social exclusion is an uncertain and complex circumstance in which the excluded individual interprets the situation and source of threat (i.e., the excluder) in dramatically different ways over time. Future research would benefit from investigating temporal change during social exclusion for a better understanding of psychological and neural correlates during social exclusion.

## Intrapersonal processes following social exclusion: social monitoring system

Once people are excluded by others, they activate an outer monitoring system, called the “*social monitoring system (SMS)*” (Pickett and Gardner, 2005). According to the conceptualization of SMS, this system enhances perceptive and cognitive responses to social cues and social information (e.g., facial expressions and vocal tone). SMS is considered an adaptive system that attunes excluded people to information that will help them navigate the social environment more successfully (e.g., Pickett et al., 2004; Gardner et al., 2005a,b; Pickett and Gardner, 2005). Supporting this supposition, prior studies have revealed that social exclusion causes increased attention and perception in response to social information (e.g., Gardner et al., 2000; Pickett et al., 2004; Bernstein et al., 2008; DeWall et al., 2009a). Although relatively few studies have focused on neural correlates of the SMS, prior studies have shown that the brain regions related to **mentalizing** and mirror neuron networks—the dorsomedial prefrontal cortex (dmPFC), temporoparietal junction (TPJ), and precentral gyrus (PG)—and ERP components related to attention and facial encoding—P1 and N170—might be involved in the SMS (e.g., Moor et al., 2012; Powers et al., 2013; Beyer et al., 2014; Kawamoto et al., 2014; Will et al., 2015). Future studies should strive toward a deeper understanding by further clarifying the neural correlates of the SMS.

KEY CONCEPT 5 **Mentalizing**

The ability to extract and understand others' thoughts and beliefs.

## Interpersonal processes following social exclusion: behaviors and self-recovery

Table 2 summarizes the key studies that have investigated the role of interpersonal processes of social exclusion. Social exclusion not only shifts an individual's cognitive responses but also influences him to change his behaviors in an attempt to regain social acceptance and/or to avoid further social exclusion. Previous findings have indicated that social exclusion causes both **prosocial** and **antisocial behaviors** (e.g., Twenge et al., 2001; Warburtona et al., 2006; Maner et al., 2007; Chow et al., 2008; DeWall et al., 2009b; Wesselmann et al., 2010; DeWall and Bushman, 2011; DeWall and Richman, 2011; Kawamoto et al., 2013a, 2014). *Social reconnection theory* proposes that excluded people behave prosocially only in the presence of cues that promise transfer of social affiliation (Maner et al., 2007). Supporting this notion, prior studies have revealed that people behave prosocially following social exclusion even when they experience cues that indicate only a remote possibility of social affiliation (e.g., Maner et al., 2007; Lakin et al., 2008; Kawamoto et al., 2014). For example, prior findings have indicated that excluded individuals show heightened willingness to make new friends via student services (Maner et al., 2007), increased behavioral mimicry of a new interaction partner (Lakin et al., 2008), and enhanced facial mimicry in response to pictures of strangers' smiles (Kawamoto et al., 2014). In addition, social exclusion induces increased progesterone production—a hormone that reflects an individual's motivation to affiliate (Frye et al., 2000)—when excluded individuals anticipated interaction with a new group (Maner et al., 2010). Thus, excluded people behave prosocially if there are cues of social acceptance—typically toward those who are not involved in the social exclusion.

**Table 2:** **Key studies on interpersonal processes of social exclusion**.

**References**	**Tools**	**Key findings**
Chester et al., 2014	fMRI	People high in executive function showed a negative relationship between dACC activation during social exclusion and aggression following social exclusion, whereas those low in executive function showed the opposite pattern.
DeWall et al., 2011		Social exclusion triggered an automatic emotional regulation process in which positive emotions were highly accessible.
Kawamoto et al., 2014	ERP and EMG	Excluded individuals showed enhanced facial mimicry (i.e., zygomaticus major activity) in response to smiling faces.
Lakin et al., 2008		Excluded individuals showed enhanced behavioral mimicry.
Maner et al., 2007		Excluded individuals showed enhanced prosocial behaviors in response to people who promised social affiliation.
Riva et al., 2015	tDCS	tDCS stimulation to rVLPFC decreased aggression following social exclusion.
Twenge et al., 2001		Social exclusion caused aggression.
Zadro et al., 2006		Excluded people reported identical levels of fundamental needs as compared to those who were included after a 45-min delay. Excluded people with high social anxiety reported decreased levels of fundamental needs relative to those with low social anxiety after a 45-min delay.

KEY CONCEPT 6 **Prosocial behavior**

Behaviors performed with the intention of helping others and being included by others. Social exclusion studies deal with prosocial behavior across a wide range, such as willingness to participate in a group, voting, relational evaluation, and behavioral mimicry.

KEY CONCEPT 7 **Antisocial behavior**

Behaviors performed with the intention of harming others. Social exclusion studies typically refer to antisocial behavior as aggression. Aggression is measured by highly valid methods such as the volume of loud white noise and amount of hot spicy sauce given to others.

In contrast, excluded individuals tend to behave antisocially toward their excluder. Previous studies have revealed that social exclusion causes aggression toward the excluder (e.g., Buckley et al., 2004; Chow et al., 2008; Kawamoto et al., 2013a). Thus, excluded individuals seem to behave aggressively rather than prosocially toward others that convey cues of social threat. Furthermore, excluded individuals lash out at others those who are not involved in social exclusion (e.g., Gaertner et al., 2008; DeWall et al., 2009b; Wesselmann et al., 2010). How can we buffer the influence of social exclusion on aggressive behavior? Previous research has sought after variables that moderate the link between social exclusion and aggression. For instance, a previous study indicated that individual differences in executive function—cognitive ability that regulates goal oriented behaviors—and dACC activity in response to social exclusion interacted to predict the relation between social exclusion and aggressive behavior (Chester et al., 2014). This study indicated that people with better executive function are less aggressive when they feel social pain (i.e., greater dACC and AI activity), whereas those with poor executive function are more aggressive. In addition, recent studies have revealed that stimulation of the right VLPFC by a transcranical direct current (tDCS) during social exclusion reduced social pain (Riva et al., 2012), and aggressive behavior following social exclusion (Riva et al., 2015). Thus, trait and situational behavioral and emotional regulation seem to play a key role in reducing aggressive behavior following social exclusion.

Excluded people seem to be able to recover from the aversive impact of social exclusion without any direct interaction with others (e.g., prosocial or antisocial behavior toward others). A prior study found that after a 45 min delay, socially excluded participants recovered their primary needs, including the sense of self-esteem, belonging, control, and meaningful existence (Zadro et al., 2006). This finding implies that people have a self-recovery system that can effectively buffer against the aversive influences of social exclusion. One possible mechanism of this system is that excluded people use an inner representation of social connection (e.g., memories of their family, mental images of favorite characters from novels). According to the *belonging regulation model*, people use indirect strategies to regain a sense of social connection when they feel unconnected (Gardner et al., 2005b). Supporting this notion, previous studies have implied that any entity with which an individual can feel a social connection—a god, comfort food, or a favorite television character—could diminish the aversive impacts of social exclusion (e.g., Derrick et al., 2009; Troisi and Gabriel, 2011; Laurin et al., 2014). In addition, excluded individuals set in motion an automatic emotion regulation process in which positive emotions become highly accessible, which relates to positive mental health (DeWall et al., 2011). Thus, excluded individuals seem to deal flexibly with their mental representations and social environment to regulate and regain the feeling of social connection.

## Social exclusion as stored experience: from the perspective of individual differences

Table 3 summarizes the key studies that have investigated individual differences in intrapersonal and interpersonal processes of social exclusion. There are individual differences in both intrapersonal and interpersonal processes of social exclusion (e.g., Kross et al., 2007; Maner et al., 2007, 2010; Masten et al., 2009; Onoda et al., 2010; Yanagisawa et al., 2011a,b; DeWall et al., 2012; Nakashima et al., 2013; Chester et al., 2014, 2015). For instance, prior findings have indicated that people with low self-esteem have more self-reported social pain and show increased dACC activity in response to social exclusion as compared to those with high self-esteem (Onoda et al., 2010). In addition, people with high trait rejection sensitivity and low childhood socioeconomic status exhibited reduced VLPFC activity in response to social exclusion (Kross et al., 2007; Yanagisawa et al., 2013). How are these individual differences in response to social exclusion developed?

**Table 3:** **Key studies on individual differences of intrapersonal and interpersonal processes of social exclusion**.

**References**	**Tools**	**Key findings**
Bolling et al., 2011a	fMRI	Children with autism spectrum disorder (ASD) showed less ventral ACC and insula activations during social exclusion.
Chester et al., 2015	fMRI	Alexithymia was related to less dACC activity during social exclusion.
DeWall et al., 2012	fMRI	People with high attachment anxiety showed increased dACC and AI activation, whereas individuals with high attachment avoidance showed decreased dACC and AI activation in response to social exclusion.
Kross et al., 2007	fMRI	People with high rejection sensitivity showed decreased lateral frontal cortex activation in response to social exclusion related to painting.
Masten et al., 2011	fMRI	Adolescences with ASD showed less subgenual ACC, AI, and VLPFC activation during social exclusion.
Maurage et al., 2012	fMRI	People with high alcohol dependence showed increased insula and decreased rVLPFC activation during social exclusion, and increased dACC activation during re-inclusion relative to those with low alcohol dependence.
Onoda et al., 2010	fMRI	Individuals with low self-esteem showed increased dACC activation in response to social exclusion as compared to those with high self-esteem.
Yanagisawa et al., 2011a	NIRS	People high in general trust showed increased rVLPFC activation in response to social exclusion as compared to those with low general trust.
Yanagisawa et al., 2013	NIRS	People high in childhood socioeconomic status showed increased rVLPFC activation in response to social exclusion as compared to those with low childhood socioeconomic status.

According to *optimal calibration theory*, early life history shifts one's neural responses to social exclusion (Chester et al., 2012). Specifically, Chester et al. argued that unpredictable social rejection in early life history causes a “hyper-activated” social pain system (i.e., the dACC and AI) whereas chronic social rejection results in a “deactivated” social pain system. Supporting this notion, a prior study indicated that people with high attachment-anxiety—reflecting higher unpredictable social rejection—showed increases in both dACC and AI activity whereas people with high attachment-avoidance—reflecting higher chronic social rejection—showed decreases in dACC and AI activity in response to social exclusion (DeWall et al., 2012). In addition, a recent study revealed that having a history of chronic victimization increased the magnitude of cardiovascular blunting in response to social exclusion (Newman, 2014), further supporting the notion that past social exclusion experiences carry over to influence responses to future social exclusion. Thus, past social exclusion experiences are possibly stored with individuals' experiences in memory, resulting in an effect on their responses to future social exclusion.

Studies have also been conducted that have investigated intrapersonal and interpersonal processes of social exclusion in clinical populations (e.g., Sebastian et al., 2009; Bolling et al., 2011a; Masten et al., 2011; McPartland et al., 2011; Maurage et al., 2012; Domsalla et al., 2014). One such study for example, has indicated that highly alcohol dependent people have increased insula and decreased rVLPFC activation during social exclusion relative to less alcohol dependent people (Maurage et al., 2012). In addition, highly alcohol dependent people showed increased dACC activation during re-inclusion—being included after being excluded—compared to less alcohol dependent people. Another study that was designed to clarify the mechanisms of social phobia by manipulating attentional bias for threatening faces (Heeren et al., 2012) indicated that the induction of attentional bias for threatening faces resulted in increased anxiety during social exclusion. These findings imply that investigating intrapersonal and interpersonal processes of social exclusion in clinical populations could contribute to understanding the mechanisms of clinical pathologies and to develop effective intervention methods for such conditions.

## Conclusion and future directions

Over the past few decades, social exclusion has received a lot of attention especially in the social psychology and social neuroscience disciplines. Given that social exclusion is critically vital for humans, it is not surprising that social exclusion affects a wide range of our perceptions, cognitions, affect, behaviors, and psychological adaptations. Although prior studies have revealed a great deal about the intrapersonal and interpersonal processes of social exclusion, some aspects need further clarification. To conclude, we describe four directions for future research, which we believe is worthwhile investigating.

Firstly, future research should explore the constructional elements of social exclusion in more detail, since social exclusion is a complex phenomenon (e.g., Smart Richman and Leary, 2009; Williams, 2009; Cacioppo et al., 2013). In fact, peoples' perceptions, cognitions, emotions, and motivations are changed over time by social exclusion (e.g., Moor et al., 2012; Wesselmann et al., 2012b; Kawamoto et al., 2013b; Themanson et al., 2013). In addition, social exclusion has been studied in several forms, such as participants being ostracized on the computer screen (Cyberball), being told that they would end up alone (future life manipulation), being told that they were not chosen by an experimental partner (get-acquainted task), or evaluating feedback about preference from peers (rejection paradigm). Although all prior manipulations have involved threats to a sense of relational value and need for belonging (Smart Richman and Leary, 2009), differences in the manipulations should be investigated further, as recent studies and reviews have argued they may play a role in the studies' outcomes (e.g., Blackhart et al., 2009; Gerber and Wheeler, 2009; Bernstein and Claypool, 2012). For instance, a recent study found that future life and Cyberball manipulations differed in severity, with one resulting in an increase and the other a decrease in pain tolerance/threshold (Bernstein and Claypool, 2012). More specifically, the authors argued that feedback indicating a future alone was a bigger threat than exclusion in Cyberball, which resulted in decreasing pain sensitivity (numbing) whereas exclusion in Cyberball resulted in increasing pain sensitivity (hypersensitivity). In addition, a previous study indicated that exclusion from a self-resembling in-group resulted in higher dACC activity relative to exclusion from an out-group (Krill and Platek, 2009). Finally, merely being accepted by one other person during social exclusion could reduce aggressive behavior following social exclusion (DeWall et al., 2010b). Thus, intrapersonal and interpersonal processes of social exclusion could be influenced by the severity, source, and situation of social exclusion.

Secondly, future research should study the boundary and interpretation of social exclusion. Previous research has revealed that people are adept at detecting the slightest hint of social exclusion (e.g., Williams et al., 2000; Smith and Williams, 2004; Zadro et al., 2004; Gonsalkorale and Williams, 2007; Kross et al., 2007; Williams, 2009; Wirth et al., 2010; Van Beest et al., 2011), since social exclusion is quite vital for survival and reproduction (Williams, 2009; Wesselmann et al., 2012a). While it seems that accurately detecting any potential cues of social exclusion would be better than missing the cues completely, excessive misinterpretation of non-exclusive social cues would result in maladaptive outcomes. For instance, people high in trait rejection sensitivity—defined as anticipatory anxiety about, a readiness to perceive, and behavioral overreactions to social rejection—tend to interpret ambiguous situations as social rejection (Downey and Feldman, 1996), and show heightened psychological difficulties such as depression and aggression (e.g., Downey and Feldman, 1996; Downey et al., 1998, 2000; Ayduk et al., 1999, 2001; Downey and Romero-Cayas, 2005; Harper et al., 2006; Romero-Canyas et al., 2010). Thus, accurately detecting social exclusion and excessive misinterpretation of social exclusion seem to have different effects on individuals' psychological adaptation.

**Signal detection theory** (SDT: Green and Swets, 1966; Lynn and Barrett, 2014) would provide a useful framework to investigate both detection sensitivity and interpretation bias of social exclusion. In this framework, two indexes—*sensitivity* (d′) and *response criterion* (β)—are calculated independently. Sensitivity reflects the subjects' ability to discriminate noise and signal whereas response criterion reflects the subjects' overall tendency to respond to the signal, independent of whether the actual stimulus is a signal. According to both the evolutionary perspective and rejection sensitivity studies, it is predicted that having a higher detection sensitivity to social exclusion would be related to positive psychological outcomes whereas having a liberal response criterion (e.g., being prone to interpret social cues as social exclusion) is maladaptive. In addition, SDT may benefit from investigation of the pharmacological effect of acesodyne on interpersonal processes of social exclusion. Prior studies have indicated that specific analgesics—acetaminophen and marijuana—can reduce social pain (DeWall et al., 2010a; Deckman et al., 2014). SDT may promote an understanding of the pharmacological effect of acesodyne on excluded individuals, such as whether it influences an individual's sensitivity or response criterion to social exclusion. These studies would be beneficial for developing effective intervention methods.

KEY CONCEPT 8 **Signal detection theory**

Psychophysical theory that explains the circumstances needed to distinguish signal from noise. In the framework of SDT, two parameters—sensitivity and response criterion—are estimated independently. SDT has been applied across a wide range of studies including memory, facial recognition, pain, and aggression.

Thirdly, in this article we focused mainly on specific responses to social exclusion (e.g., social pain, prosocial behavior, antisocial behavior), however, there is also a need to develop new methods to assess the reactivity to social exclusion, in order to better understanding abnormal responses of clinical populations. For example, a recent study focused on how acoustic responses (i.e., vibrations of the vocal folds during phonation and speech) following social exclusion are modulated by social anxiety (Gilboa-Schechtman et al., 2014). They found that individuals high in social anxiety showed decreased vocal confidence when reading command sentences following social exclusion, whereas those low in social anxiety showed the opposite pattern. Reactions that match specific psychopathologies would help us to better understand the mechanisms of such pathologies, as well as to develop effective interventions.

Finally, it is important to focus on not only targets of social exclusion, but also on the sources of social exclusion. There is a need to investigate the nature and the consequences of the act of excluding, in order to better understand social exclusion (Zadro and Gonsalkorale, 2014). In this focused review, we have mainly focused on targets of social exclusion, and positioned social exclusion at the top of our model. Although some studies have investigated the nature and consequences of the act of excluding (e.g., Poulsen and Kashy, 2012; Bastian et al., 2013; Legate et al., 2013), most social exclusion studies have focused only on the targets of exclusion. Examining the nature and consequence of sources of social exclusion, as well as the interplay between targets and sources would not only help to better understand the nature of social exclusion, but would also have important implications for understanding intimate relationships.

In conclusion, we have provided an integrative framework of the intrapersonal and interpersonal processes of social exclusion, and summarized the findings of prior studies that have made important contributions to understanding what happens during and after social exclusion. We hope that our review and framework provide an effective approach for further understanding the effects of social exclusion on intrapersonal and interpersonal processes.

### Conflict of interest statement

The authors declare that the research was conducted in the absence of any commercial or financial relationships that could be construed as a potential conflict of interest.
